# Hydrothermally Assisted Conversion of Switchgrass into Hard Carbon as Anode Materials for Sodium-Ion Batteries

**DOI:** 10.1021/acsami.4c02734

**Published:** 2024-05-23

**Authors:** Yilin Li, Dawei Xia, Lei Tao, Zhiyuan Xu, Dajun Yu, Qing Jin, Feng Lin, Haibo Huang

**Affiliations:** 1 Department of Food Science and Technology, Virginia Tech, 1230 Washington Street SW, Blacksburg, Virginia 24061, United States; 2 Department of Chemistry, Virginia Tech, 1040 Drillfield Drive, Blacksburg, Virginia 24061, United States; 3 School of Food and Agriculture, University of Maine, 5763 Rogers Hall, Orono, Maine 04469, United States

**Keywords:** switchgrass, hard carbon, sodium ion batteries, hydrothermal pretreatment, anode materials, lignocellulosic biomass

## Abstract

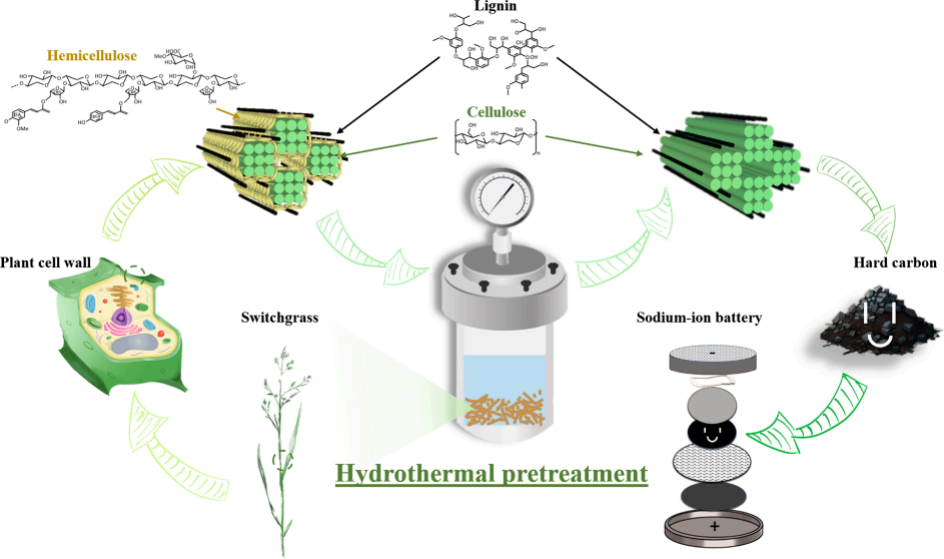

Sodium-ion batteries (SIBs) are emerging as a viable alternative to lithium-ion batteries, reducing the reliance on scarce transition metals. Converting agricultural biomass into SIB anodes can remarkably enhance sustainability in both the agriculture and battery industries. However, the complex and costly synthesis and unsatisfactory electrochemical performance of biomass-derived hard carbon have hindered its further development. Herein, we employed a hydrothermally assisted carbonization process that converts switchgrass to battery-grade hard carbon capable of efficient Na-ion storage. The hydrothermal pretreatment effectively removed hemicellulose and impurities (e.g., lipids and ashes), creating thermally stable precursors suitable to produce hard carbon via carbonization. The elimination of hemicellulose and impurities contributes to a reduced surface area and lower oxygen content. With the modifications, the initial Coulombic efficiency (ICE) and cycling stability are improved concurrently. The optimized hard carbon showcased a high reversible specific capacity of 313.4 mAh g^–1^ at 100 mA g^–1^, a commendable ICE of 84.8%, and excellent cycling stability with a capacity retention of 308.4 mAh g^–1^ after 100 cycles. In short, this research introduces a cost-effective method for producing anode materials for SIBs and highlights a sustainable pathway for biomass utilization, underscoring mutual benefits for the energy and agricultural sectors.

## Introduction

Lithium-ion batteries (LIBs) have dominated the mobile energy storage market over the past few decades.^1^ Nevertheless, the application of LIBs in large-scale electrical energy storage remains constrained by the high cost of lithium and supply chain risks associated with critical materials like nickel and cobalt.^2^ To address these challenges, research has pivoted toward developing alternative batteries based on more earth-abundant elements, such as sodium, potassium, and zinc.^3−5^ Of these, sodium-ion batteries (SIBs) stand out as a particularly viable candidate for future large-scale energy storage solutions due to their decent energy density and the abundant availability of sodium.^6^

Developing high-performance cathode and anode materials is crucial for the commercial viability of SIBs. Recent advancements in cathode materials, including layered transition-metal oxides, polyanionic compounds, and Prussian blue analogs, have demonstrated remarkable successes.^7,8^ However, finding suitable anode materials for SIBs remains a challenge. Graphite, the predominant anode material in LIBs, cannot efficiently store Na-ions due to thermodynamic prohibition of Na-graphite compound formation.^9,10^ Consequently, research has pivoted toward alternative anode materials like disordered carbon, with hard carbon from biomasses being extensively studied for its advantageous amorphous structure and expansive interlayer spacing.^11^ A wide range of biomasses, including lotus stem, orange peel, peanut shell, tea leaves, lignin, basswood, and brewer’s spent grain, have been utilized as hard carbon precursors.^12−18^ Despite this, the storage capacity of sodium ions in biomass-derived hard carbon is insufficient, and many of these materials exhibit poor cycling stability and low initial Coulombic efficiency (ICE). Moreover, the irregular structure of hard carbons tends to associate with the occurrence of irreversible adsorption of Na-ions, leading to capacity fading and reduced battery performance over time.^19^

Previous studies employed an activation process to enhance the properties of the synthesized hard carbon, aiming to increase the initial reversible capacity and improve the cycle stability.^20−22^ However, the persistently low ICE of hard-carbon anodes remains a main bottleneck.^19,23^ In addition, this activation process often involves the use of strong acids or bases, such as HCl and KOH, which not only raises environmental concerns but also escalates material and processing expenses.^20,24^ Hydrothermal pretreatment is an environmentally friendly process to convert various biomass into value-added biochemicals.^25^ The process operates at 150–260 °C under high pressures, where water medium is pressurized to sub- and supercritical water, functioning as a solvent, reactant, and catalyst.^26^ Compared to chemical activation methods, hydrothermal pretreatment stands out as a promising technique due to its simplicity, efficiency, reduced time requirement, and most importantly without relying on environmentally detrimental chemicals.^15,27−29^ Previous studies have indicated that hydrothermal pretreatment can effectively modulate the carbon structure and improve the electrochemical performance of hard carbon materials when used before carbonization.^6,30^ However, the precise impact of key hydrothermal processing parameters, particularly the pretreatment temperature, on the structural properties of the resulting hard carbon and the sodium ion storage remains unclear.

The objective of this study is to develop a hydrothermally assisted carbonization process to convert switchgrass into hard carbon as an anode material in SIBs. Particularly, the influence of hydrothermal temperature on hard carbon from switchgrass was investigated, aiming to determine the optimal operating condition for pretreating switchgrass as a suitable hard carbon precursor. Switchgrass is an appealing choice as the raw biomass material for producing hard carbon due to its low cost, high biomass yield, and remarkable adaptability to a variety of growth environments. Most importantly, its high cellulose and lignin content makes it suitable to produce hard carbons with a high yield and a stable structure.^31,32^ Recognizing these intrinsic qualities of switchgrass, our research leverages its potential, setting the stage for a novel approach to biomass valorization by converting them into high-value energy storage materials.

## Materials and Methods

### Materials

Switchgrass cultivar Alamo × Dacotah was obtained from the School of Plant and Environmental Sciences at Virginia Tech (Blacksburg, VA, USA). Chemicals including isopropyl alcohol (IPA), ethanol, sulfuric acid, barium carbonate, potassium bromide, ethylene carbonate (EC) (99%), and sodium trifluoromethanesulfonate (NaOTF) (>98%) were purchased from Fisher Scientific (Fair Lawn, NJ, USA). d-(+)Glucose, d-(+)xylose, d-(+)arabinose, d-(+)galactose, *N*-methyl-2-pyrrolidone (NMP), diglyme (>99%), sodium biphenyl, tetrahydrofuran (THF), sodium hexafluorophosphate (NaPF_6_), dimethyl carbonate (DMC) (99%), and dimethyl ether (DME) were purchased from Sigma-Aldrich (St. Louis, MO, USA). Cytiva Whatman filter paper (Grade 50, CAT No. 1450-090) and a glass microfiber prefilter (glass fiber, GF/D) were purchased from Fisher Scientific (Fair Lawn, NJ, USA). Polyvinylidene fluoride (PVDF) binder was purchased from MTI Corporation (Richmond, CA, USA).

### Synthesis of Hard Carbon

**Figure 1:**
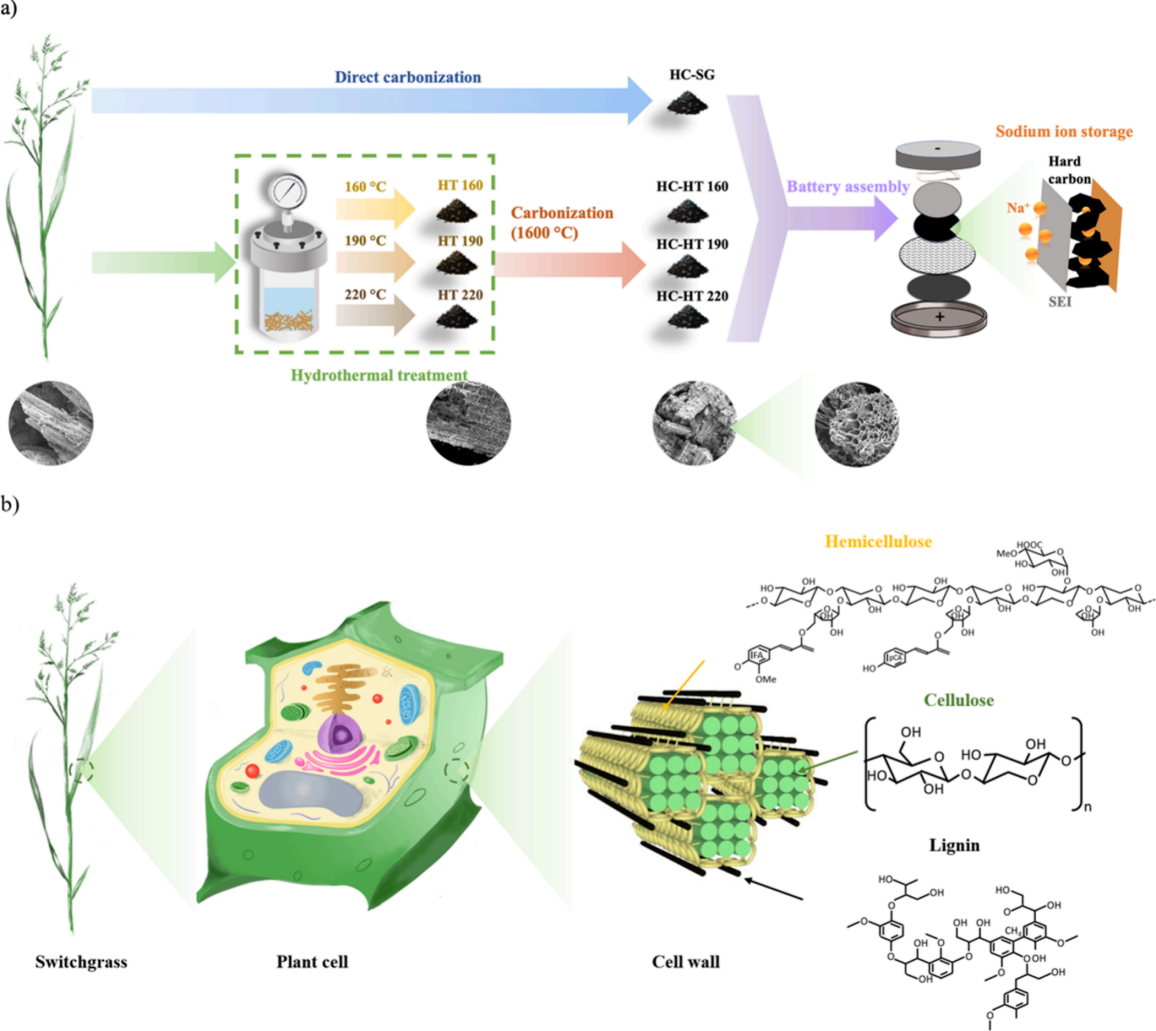
(a) Schematic illustration of the synthesis routes of switchgrass-derived hard carbons. The switchgrass is subjected to hydrothermal pretreatment at three different temperatures (i.e., 160, 190, and 220 °C), followed by carbonization to produce hard carbon, named HC-HT160, HC-HT190, and HC-HT220. For comparison, the switchgrass is directly carbonized to hard carbon, denoted as HC-SG. (b) General structure of switchgrass cell wall matrix and the major components (i.e., cellulose, hemicellulose, and lignin) and structure of switchgrass. The hydrothermal pretreatment modifies the structure of the precursor by removing hemicellulose and impurities.

As illustrated in Figure 1a, a hydrothermal pretreatment was applied to modulate the chemical and structural properties of switchgrass, which was subsequently carbonized to produce hard carbon. The process started with grinding the dried switchgrass to 1 mm powders using a hammer mill, followed by mixing the resultant powders with deionized water at a mass ratio of 1:10. The mixture then underwent hydrothermal pretreatment in a 600 mL stainless steel reactor (Parr Instrument Company, Moline, IL, USA) with continuous stirring at 40 rpm. The reactor was sealed and subjected to consistent reaction temperatures of 160 °C, 190 °C, and 220 °C, each for 12 h. Following this, the resultant carbon-rich solid, i.e., hydrochar, and liquid byproduct were separated using a filtration setup comprising a flask and cotton filter paper. The separated hydrochars, denoted as HT160, HT190, and HT220, were oven-dried at 80 °C overnight. These dried hydrochars were then carbonized at 1600 °C for 2 h in a tube furnace (CM 1700 series, CM Furnaces Inc., Bloomfield, NJ, USA) under an N_2_ flow to produce hard carbon. The resulting hard carbons, derived from the various hydrochars and denoted as HC-HT160, HC-HT190, and HC-HT220, were compared to switchgrass carbonized directly at 1600 °C for 2 h, referred to as HC-SG.

### Characterization of Materials

The morphology of the switchgrass-derived carbon materials was visualized by using a scanning electron microscope (SEM) (JEOL IT-500HR, JEOL, Tokyo, Japan) and a transmission electron microscope (TEM) (JEOL S/TEM 2100, JEOL, Tokyo, Japan). Structural properties of the carbon materials were characterized by Raman spectroscopy with spectra collected from an XploRA PLUS Raman spectrometer (HORIBA Scientific, Tokyo, Japan) using a 532 nm laser. Polymorphism of the carbon materials was characterized by wide-angle X-ray diffraction (XRD) patterns using a SolariX system (Bruker, Billerica, MA, USA) with a Cu Kα radiation source (wavelength λ = 1.5406 Å), scanning from 18° to 52° at 0.035°/min increment. Specific surface area was calculated by the Brunauer–Emmett–Teller (BET) method using a gas sorption analyzer (Autosorb-iQ, Quantachrome Instruments, Anton Paar Quanta Tec Inc., Boynton Beach, USA) at −196 °C after an outgas process at 200 °C for 12 h. Pore distribution was determined from the N_2_ adsorption isotherms following the Barrett–Joyner–Halenda (BJH) method. X-ray photoelectron spectroscopy (XPS) was conducted by using a scanning photoelectron spectrometer microprobe (PHI Quantera SXM, ULVAC-PHI Inc., Japan), utilizing an Al anode as the monochromatized X-ray source to quantitatively analyze the chemical elements and chemical states of the surface. More experimental details of characterization of switchgrass and hydrochars are provided in the Supporting Information.

### Electrochemical Measurements

The hard carbon anode was fabricated by mixing hard carbon, carbon black, and PVDF binder in a mass ratio of 90:2.5:7.5. The mixture was dispersed in NMP solvent, cast onto Cu foil, and then dried thoroughly in a vacuum oven. The resulting anode was then assembled in a coin cell battery with a sodium foil as the counter electrode, a glass fiber (GF/D 47, Whatman) as the separator, a spacer, a spring, and 1 M NaOTf in diglyme as its electrolyte. The half-cell battery test was conducted over a voltage range of 0.001–3 V (vs Na^+^/Na) at 0.1 A/g. In full-cell battery configuration, the Na_3_V_2_(PO_4_)_3_ (NVP) cathode was fabricated by mixing active material, carbon black, and PVDF binder in a mass ratio of 90:5:5, casting on aluminum foil. Hard carbon anode was prepared with presodiation to compensate the irreversible sodium ion loss in full-cell SIBs. Presodiation was conducted by immersing the anode films in 2 mL of 0.3 M sodium biphenyl in THF at the controlled reaction time in the glovebox. Then, the sodiated electrode was washed with DME and dried naturally in the glovebox. Full cell was then assembled using NVP cathode, 60 μL of electrolyte (1 M NaOTf diglyme), glass fiber separator, and the presodiated anode. Full cell has a voltage window of 1.8–3.8 V and N/P ratio of 1.1. All batteries were assembled in an argon-filled glovebox. A constant-current charging and discharging test was carried out on a Landt battery test system (CT3002A, Landt Instruments, Wuhan, China). Cyclic voltammetry (CV) tests were conducted at scan rates of 0.1–1 mV/s using a multichannel potentiostat-galvanostat. The galvanostatic intermittent titration technique (GITT) was performed by alternately charging/discharging using a pulse current at 0.05 A/g for 20 min and relaxation for 4 h until the potential achieved 0.001 V vs Na^+^/Na. One formation cycle at 0.05 A/g was applied before GITT measurement.

## Results and Discussion

### Characterization of Switchgrass and Hydrochar

**Figure 2:**
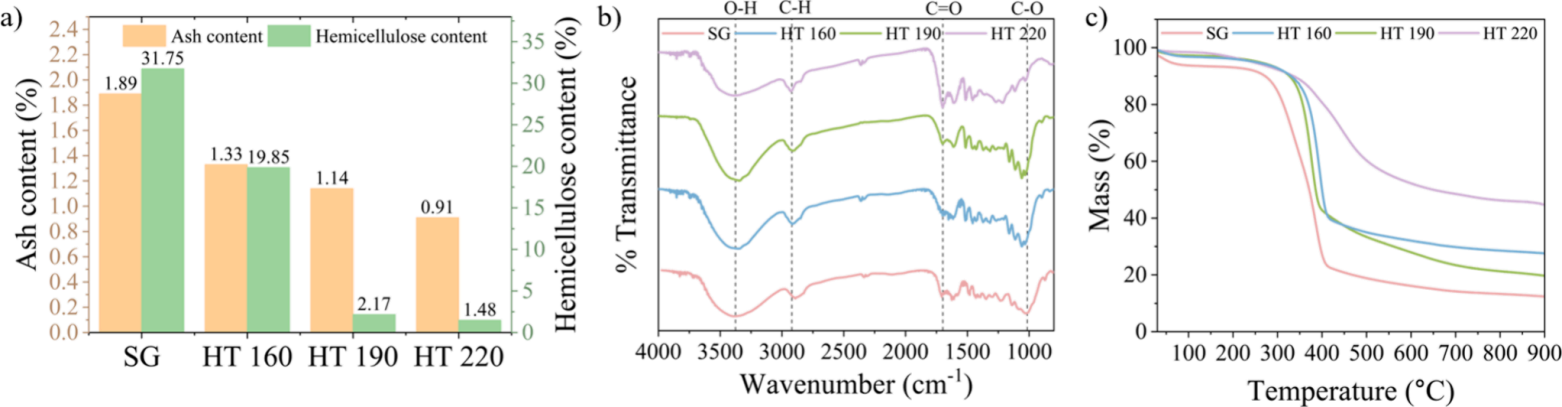
Detailed overview of the material characterizations conducted on switchgrass and hydrochars, illustrating the chemical and physical properties: (a) hemicellulose and ash content, highlighting the effect of hydrothermal pretreatment on precursor’s chemical composition; (b) FTIR spectra in the wavenumber range of 4000–400 cm^–1^; (c) TGA, from 30 to 900 °C with a ramp temperature of 5 °C/min, showing the material’s thermal stability and decomposition profile.

A few pioneering studies have reported that the physicochemical and electrochemical properties of the biomass-derived carbon materials can be significantly impacted by the different chemical composition of the precursors and different processing routes to pretreat the biomass.^15,34^ Chemical composition analysis revealed that raw switchgrass contains 38.5% cellulose, 31.8% hemicellulose, and 16.1% lignin, which together constitute 86% of its total dry weight. The general structure of the switchgrass cell wall is shown in Figure 1b. Additionally, it contains minor constituents, such as ash (minerals), protein, and lipid (Table S1). The hydrothermal pretreatment significantly reduced hemicellulose and other minor constituents. For instance, the hemicellulose content decreased to 9.9% and the ash content decreased to 1.3% in the switchgrass pretreated at 160 °C (HT160) (Figure 2a). When the pretreatment temperature increased to 220 °C, the hemicellulose content further decreased to 1.5% and the ash content decreased to 0.9%. Each type of biomass contains a certain amount of ash, primarily from the structural ash found within the cross-linked structure of biomass, as well as from soil contamination and loose dirt accumulated during harvesting.^35,36^ Ash from soil contamination and loose dirt that cling onto biomass was removed by hot water flow during hydrothermal pretreatment at mild temperatures. Additionally, increased acidity due to the release of acetal groups from biomass during the hydrothermal pretreatment can potentially solubilize inorganic minerals. These explain the ash reduction in all hydrochar samples. As the hydrothermal temperature exceeded 200 °C, the hemicelluloses were mostly solubilized, and the cellulose started reacting.^37^ The high temperature broke down the lignocellulosic matrix, potentially releasing ash entrapped within the cross-linked structure.^36,38^ Thus, the ash content decreased with an increasing pretreatment severity. With more hemicellulose and impurities removed, the morphology of the precursors became noticeably rougher (Figure S1). Previous research suggests that hemicellulose and other impurities (e.g., ash, lipid) in raw biomass adversely affect the electrochemical performance of derived hard carbon anodes, including a low specific capacity, reduced cycling stability, increased irreversible capacity loss, and a poor rate capability.^15,39^ Conversely, cellulose content is positively correlated with the anode’s specific capacity and cycling stability.^15,19^ Therefore, applying hydrothermal pretreatment to switchgrass to remove hemicellulose and other impurities can potentially enhance its viability for the synthesis of high-quality hard carbon production.

FTIR analysis was employed to elucidate the chemical structure and functional groups present on the surface of switchgrass and its derived hydrochars. The characteristic functional groups, along with their possible explanations, are delineated in Table S2. The FTIR spectra, shown in Figure 2b, generally exhibited peaks at similar locations but with differences in peak shapes and intensities across samples, indicating chemical composition changes induced by the hydrothermal pretreatment. One critical peak, 3400 cm^–1^, indicative of O–H stretching, revealed the presence of cellulose, hemicellulose, and lignin. Notably, this peak in hydrochar prepared at 220 °C (HT220) appeared broader and less intense, mainly due to the effective removal of hemicellulose. Concurrently, the peak at around 1060 cm^–1^, associated with C–O stretching in hemicellulose and cellulose, attenuated in HT220, further corroborating the decomposition of hemicellulose. Additionally, an intensified peak around 1700 cm^–1^ in HT220s spectrum, corresponding to the C=O stretching, suggests a relative increase in cellulose and lignin content, likely due to the concurrent removal of hemicellulose and other impurities (e.g., protein, fat, ash). These spectral findings, in alignment with the chemical composition analysis, reinforce that hydrothermal pretreatment is effective in removing hemicellulose and other minor components.

Thermogravimetric analysis (TGA) was employed to determine the thermal decomposition characteristics of raw and pretreated switchgrass (i.e., hydrochars). The TGA data indicated that raw switchgrass had the highest mass loss among all samples, particularly between 300 and 500 °C (Figure 2c). This substantial loss is likely due to the decomposition of hemicellulose, which is more heat labile compared with cellulose and lignin. On the other hand, the hydrochars (HT160, HT190, HT220) exhibited enhanced thermal stability compared to raw switchgrass, implying the formation of a carbon matrix that is chemically stable during the hydrothermal pretreatment. Among all samples, HT220 demonstrated the greatest thermal stability, exhibiting minimal mass loss, a reflection of the reduced thermolabile hemicellulose content and a higher concentration of thermally stable cellulose and lignin. Beyond 500 °C, the analysis showed a long tail with a slow mass loss, which is probably due to the decomposition of cellulose and lignin and the formation of char. Together, the TGA findings indicate that the hydrothermal pretreated samples, especially HT220, are more thermally stable as a precursor for hard carbon production. Such stability is crucial not only because it resulted in a higher yield of hard carbon (Table S3) but also considering its impact to battery performance; the precursor with high thermal stability can promote the energy density of the produced hard carbon and directly impacts the battery’s storage capability.^40^

Overall, chemical composition analysis, FTIR, and TGA provide important insights into the physicochemical properties of switchgrass and hydrochars. Based on the results, hydrothermal pretreatment proves to be a highly effective technique for removing hemicellulose and other impurities from switchgrass. Notably, the presence of these impurities is detrimental to electrochemical performance.^41,42^ This outcome highlights the considerable potential of the hydrothermal pretreatment in manipulating the chemical composition of biomass, providing great potential to enhance the electrochemical properties of the resulting hard carbon as a battery anode.

### Characterization of Hard Carbon

**Figure 3:**
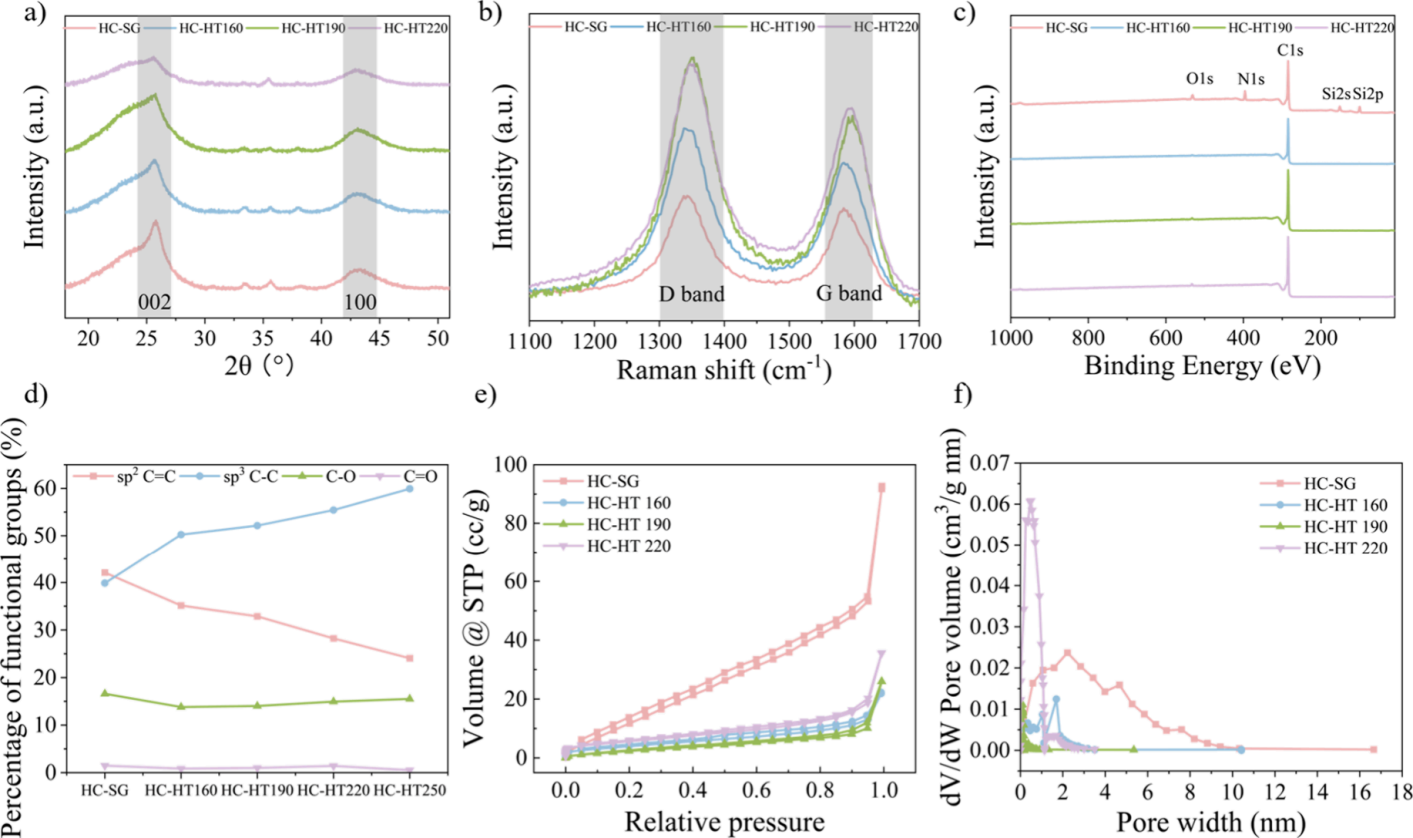
Characterization of the hard carbons: (a) XRD patterns scanned from 18° to 52° at 0.035°/min increment revealing graphitic (002) and amorphous (100) carbon peaks; (b) Raman spectra obtained using a 532 nm laser, displaying the sp^3^ D band and sp^2^ G band at 1330 and 1580 cm^–1^ respectively; (c) XPS surveys with surface elemental composition (i.e., O, N, C, and Si); (d) percentage of functional groups (i.e., C–C, C=C, C–O, and C=O) on the surface of hard carbons based on fitted XPS spectra; (e) N_2_ adsorption and desorption isotherms measured at −196 °C with (f) corresponding pore size distributions analyzed with BJH method.

This section offers a comprehensive characterization of hard carbon, aiming to elucidate the structural properties of hard carbon that dictate its electrochemical performance. The structure of hard carbon was studied with Raman spectroscopy, XRD, and XPS. The XRD patterns (Figure 3a) of hydrothermally treated hard carbons, i.e., HC-HT160, HC-HT190, and HC-HT220, displayed two broad diffraction peaks at 2θ = 25.5° and 43°, corresponding to the (002) and (100) planes, signaling an overarching amorphous carbon structure. The hard carbon from raw switchgrass, HC-SG, displayed a relatively sharper peak at 25.5°, implying more graphitic structure compared to hydrothermally pretreated counterparts. On the other hand, the hydrothermally pretreated samples had a broader peak at 25.5°, and this peak progressively broadens and decreases in intensity as the hydrothermal temperature increases from 160 to 200 °C. This is mainly because the hydrothermal pretreated sample is more thermal stable and, thus, more difficult to be decomposed and graphitized, leading to the decrease in the degree of graphitization of the produced hard carbon. The crystallite structure and size were quantified by calculating average lateral size (*L*_a_), stacking heights (*L*_c_), and interlayer spacing of (002) planes (*d*_002_) (Table S4). Intriguingly, there is no significant difference observed between hydrothermally treated and untreated carbons across these measured parameters. The alterations in these XRD patterns, i.e., the *L*_a_, *L*_c_, and *d*_002_ values, seem primarily influenced by other factors, such as carbonization temperatures, rather than the hydrothermal pretreatment.^18,43^

The Raman spectra of the produced hard carbon exhibit two distinctive peaks at approximately 1330 and 1580 cm^–1^ (Figure 3b), representing the sp^3^ carbon (D band) and the sp^2^ carbon (G band), respectively. The appearance of the D band indicates the disordered carbon structure arising from defects within the carbon lattice, while the G band represents the in-plane vibration of sp^2^ hybridized carbon atoms within ordered graphitic regions. The intensity ratio of the D band to the G band (*I*_D_/*I*_G_) quantifies the degree of disorder within the hard carbon. When compared to hard carbon derived from raw switchgrass (HC-SG), those produced from hydrothermally pretreated switchgrass exhibited a higher *I*_D_/*I*_G_ ratio. This elevation signifies a greater amount of sp^3^ carbon and a higher degree of disordered structure, indicating that the hydrothermal pretreatment plays a key role in the formation of a disordered structure within hard carbon. The higher degree of disordered structure is because the pretreatment not only decomposes the organic components of biomass, particularly the hemicellulose, but also breaks down its orderly crystalline structures. As the pretreatment temperature increased from 160 to 220 °C, the *I*_D_/*I*_G_ ratio increased, implying that the hydrothermal temperature can be used to manipulate the disordered structure of the resulting hard carbon.

The TEM images and the fast Fourier transformation (FFT) patterns of carbonized samples are illustrated in Figure S2. The TEM images exhibited disordered structures, indicating that all samples are amorphous. Untreated hard carbon (HC-SG) and hard carbon pretreated at a low temperature (HC-HT160) exhibit more ordered structures than hard carbon pretreated at higher temperatures (i.e., HC-HT190, HC-HT220). The FFT images showed no observation of diffraction patterns of crystallites, further illustrating that the samples are amorphous. The diffraction rings of HC-SG and HC-HT160 are observed to be sharper, suggesting more ordered structures within these samples. The TEM results further confirmed the conclusions from the XRD and Raman results.

The XPS analysis showed that hard carbon from raw switchgrass (HC-SG) exhibited a higher oxygen content of 5.0 atom %, in contrast to the 0.9–1.6 atom % range found in hard carbons from pretreated switchgrass (Figure 3c, Table S5). Biomass precursors inherently introduce oxygen-containing functional groups to hard carbon,^44^ which have been identified as critical factors influencing sodium storage performance in recent research.^44−46^ These oxygen-containing functional groups on hard carbon have been considered a double-edged sword in electrochemical performance for SIBs. While they can serve as active sites that enhance sodium storage capacity through redox reactions,^47^ they also intensify side reactions between carbon anode and electrolyte, accelerating electrolyte degradation and the formation of solid electrolyte interphase (SEI). Thus, reducing oxygen-containing functional groups can make hard carbon less reactive with the electrolyte, diminish electrolyte decomposition, lower the irreversible sodium ion consumption, and potentially increase the initial Coulombic efficiency (ICE).

The oxygen-containing functional groups in our carbon primarily consist of C–O–C ether and C=O carbonyl configurations (Figure 3d). The fitted XPS spectra showed that the decrease in oxygen in the pretreated hard carbons is mainly attributed to the decrease in C–O bonds (Figure 3d), which may reduce SEI formation and enhance the ICE. Moreover, the C 1s high-resolution XPS spectra (Figure S3) of the hard carbons were deconvoluted into four distinct peaks at binding energies of 284.8, 285.8, 286.4, and 287.7 eV, corresponding to C=C sp^2^, C–C sp^3^, C–O, C=O bonded carbons, respectively.^15,48^ A notable shift from raw HC-SG to HC-HT220 was observed, where the C–C sp^3^ peak grows from 39.9% to 55.4% at the expense of the C=C sp^2^ form (Figure 3d). This change underscores the impact of hydrothermal pretreatment in promoting a less graphitic carbon structure. The XPS results, together with the *I*_D_/*I*_G_ in Raman spectra, strengthen the evidence that hydrothermal pretreatment plays a crucial role in controlling the structural characteristics of the obtained carbon materials.

N_2_ adsorption–desorption isotherms were employed to examine the surface area and porosity of hard carbons (Figure 3e, Figure S4). As summarized in Table 1, untreated hard carbon (HC-SG) showed the highest specific surface areas (*S*_BET_) (67.4 m^2^/g) compared to the hydrothermally treated hard carbons (<20 m^2^/g). The pore size distribution, determined by the BJH method, reveals that the larger *S*_BET_ of HC-SG is attributed to a combination of micro- and mesopores, whereas the treated hard carbons are primarily dominated by micropores under 2 nm (Figure 3f). A large specific surface area is known to be strongly correlated with SEI formation, which results in the irreversible entrapment of sodium ions and a low ICE.^43^ Previous studies have reported that natural impurities in biomass can induce to a larger specific surface area through the self-activation during carbonization.^19,49^ In our study, the hydrothermal pretreatment effectively removed mineral impurities in biomass, consequently yielding high-purity hard carbon with a lower surface area and more uniform particle size. Increasing hydrothermal temperature from 160 to 220 °C leads to a slight increase in the *S*_BET_, probably due to the reduced pore size (Figure 3f). Thus, we conjecture that hydrothermal temperature has impact on the surface area and pore sizes of hard carbons, but other parameters such as carbonization temperature can also be influential.^6,18,50^ Via meticulously modulating both hydrothermal and carbonization temperatures, we can finely tune the specific surface area and porosity of battery-grade hard carbons.

**Table 1:** Physical Parameters and Electrochemical Properties for the Obtained Hard Carbons

sample	*S*_BET_ (m^2^/g)	*d*_002_ (Å)	*I*_D_/*I*_G_	ICE (%)	SpeCap_charge_ (mAh g^–1^)
HC-SG	67.37	3.45	1.47	75.91	235.5
HC-HT160	13.28	3.46	1.60	80.00	262.1
HC-HT190	15.95	3.46	1.71	79.60	287.5
HC-HT220	19.96	3.48	1.73	84.84	313.4

To summarize, the hydrothermal pretreatment has been found to efficiently remove hemicellulose and impurities and prestabilize the morphologies and structures of produced hard carbons. The resulting hard carbons are characterized by a higher degree of disordered structures, more micropores with a reduced specific surface area, and less extrinsic oxygen doping on the surface. These characterizations provide valuable insights into the role of hydrothermal pretreatment in the physicochemical properties of biomass-derived hard carbons. Consequently, the variations in the electrochemical performance of the produced SIBs can be reliably ascribed to the compositional, morphological, and structural properties of carbon materials.

### Electrochemical Performance of Half and Full Cell SIBs

**Figure 4:**
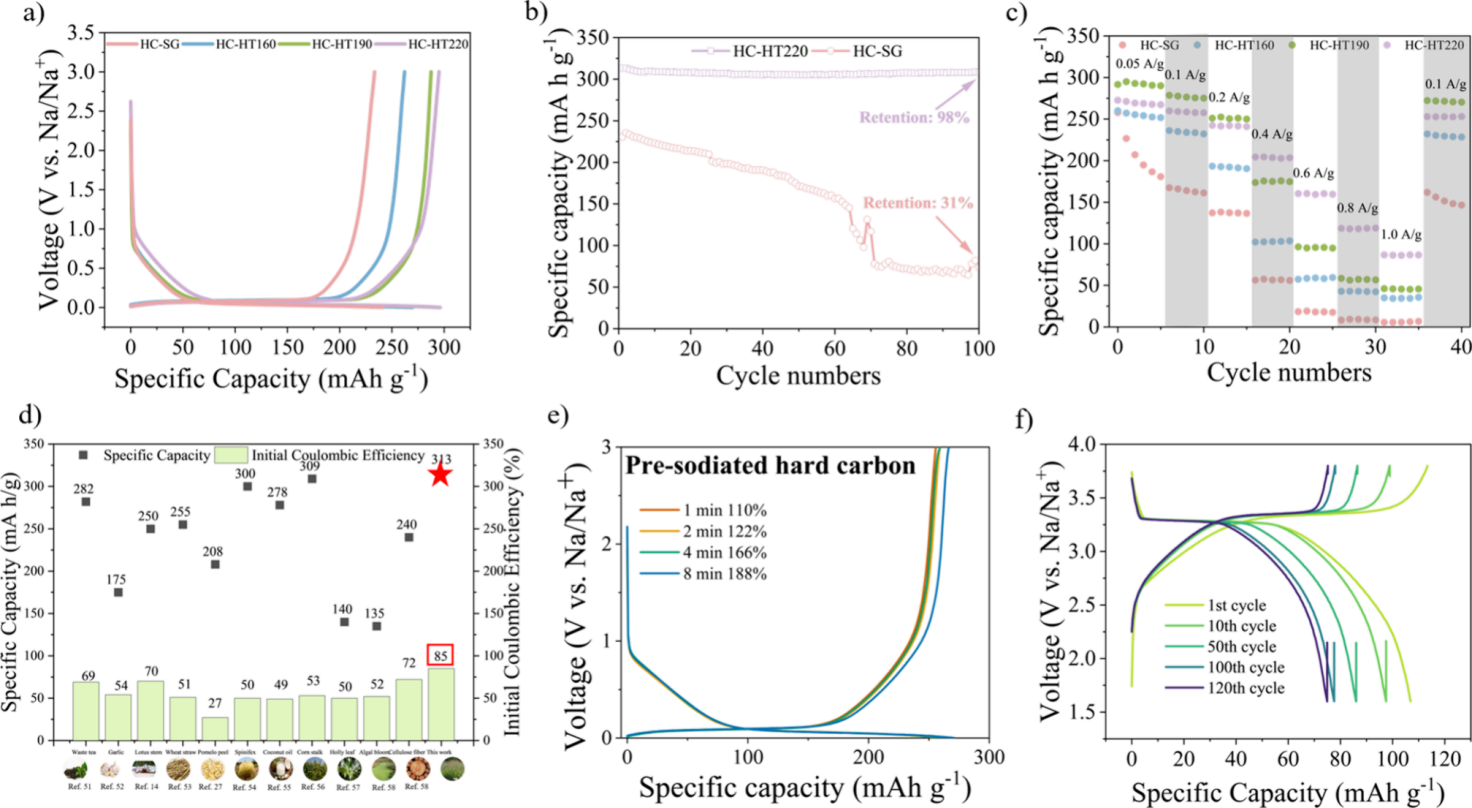
Electrochemical performance. (a) second cycle of galvanostatic charge/discharge (GCD) curves of HC-SG, HC-HT160, HC-HT190, and HC-HT220 at 0.1 A g^–1^ within the potential range of 0.001–3 V versus Na/Na^+^ using 1 M NaOTF in diglyme; b) cycling performance at 0.1 A g^–1^, of HC-SG and HC-HT220 after 100 charging–discharging cycles; (c) rate performance of HC-SG, HC-HT160, HC-HT190, and HC-HT220 at the current densities of 0.05, 0.1, 0.2, 0.4, 0.6, 0.8, and 1.0 A g^–1^ for 5 cycles each; (d) specific capacities and initial Coulombic efficiency of HC-HT220 in comparison to reported biomass-derived carbon anodes for SIBs; (e) GCD curves of hard carbon with different presodation time; (f) GCD curves of the presodiated full-cell battery using the HC-HT220 anode at 0.1 A g^–1^ after 1, 10, 50, 100, and 120 cycles.

The electrochemical testing in a half-cell configuration was employed to investigate the storage capability of the obtained hard carbons. The second cycles of galvanostatic charge/discharge (GCD) curves of hard carbons were compared, as shown in Figure 4a. The charge capacity of HC-SG was 233.4 mAh g^–1^ at the current density of 100 mA g^–1^. In contrast, the hydrothermally treated hard carbons showed significantly higher reversible charge capacities in both the sloping and plateau regions. Among all hydrothermally treated hard carbons, HC-HT220 delivered the highest charge capacity of 313.4 mAh g^–1^. This enhanced reversible capacity is mainly attributed to its most disordered carbon structure. The capacity increases in the sloping regions are strongly associated with the adsorption of sodium ions at defective sites, which is significantly influenced by the degree of graphitization. This finding agrees well with those of previous studies.^6,19^

Low ICE has been considered as the main bottleneck impeding the commercial application of hard carbon. This deficiency is largely caused by SEI formation, which results in an irreversible capacity loss during the first cycle.^19^ As shown in Table 1, the hard carbon derived from raw switchgrass (HC-SG) has a low ICE of 75.9%. On the other hand, the hydrothermally pretreated hard carbons achieved ICEs above 80%, with HC-HT220 showing the highest of 85%, which outperforms many of its biomass-derived counterparts (Figure 4d).^51−58^ The variation in ICE can be attributed to multiple factors, including (1) the adsorption of sodium ions at surface of open pores, which is influenced by specific surface area of hard carbons,^23^ (2) the formation of metallic sodium clusters due to uneven pore distribution and large pore size,^59,60^ and (3) irreversible adsorption of sodium ions at oxygen-rich sites. We infer that the higher irreversible capacity in our pretreated hard carbon, especially HC-HT220, is due to its reduced specific surface area and low oxygen content (as shown in Figure 3), all of which suppress the generation of excessive SEI and sodium consumption.

In terms of cycling performance, HC-SG shows a maximum capacity of 235.5 mAh g^–1^ in the second cycle, and the capacity rapidly drops with a high decay rate each cycle. Following 100 charge–discharge cycles, HC-SG manifests a low-capacity retention of 31.3% (Figure 4b). In contrast, the hydrothermally treated hard carbons exhibit a steadier cycling performance (Figure S5). For example, the capacity of HC-HT220 merely decreased from 313.4 to 308.4 mAh g^–1^ after 100 cycles, referring to a high-capacity retention of 98.4% and a low average decay rate of 0.0002% per cycle (Figure 4b). We also observed the exceptional cycling stability of this sample when it was tested with different electrolytes (Figure S6). The discrepancy in cycling stability can be reliably correlated to the structure of carbon materials. The pretreated hard carbons exhibit more stable structures with fewer heteroatomic doping on the surface, which minimizes side reactions with the electrolyte. The advantageous properties of the HC-HT series can be traced back to the successful elimination of thermolabile hemicellulose and other organic components from the precursors, thereby enhancing their stability and performance in SIBs. This is consistent with the previous finding that a combination of cellulose and lignin contribute to the optimal electrochemical performance.^19^

Figure 4c shows the rate performance of obtained hard carbons at different rates from 50 mA g^–1^ to 1 A g^–1^. HC-SG shows a reversible capacity of 226.7, 167.4, 137.2, 56.3, 18.4, 8.2, and 5.5 mAh g^–1^ at 0.05, 0.1, 0.2, 0.4, 0.6, 0.8, and 1 A g^–1^ with the retention of the capacity of 161.9 at 100 mAh g^–1^. HC-HT series offer a vastly superior rate capability when compared to HC-SG. While HC-HT160 exhibits a slightly lower capacity than HC-HT190 and HC-HT220 at low current densities, this difference becomes more pronounced at higher current densities. These observations suggest that the removal of hemicellulose can contribute to an improved carbon structure and thus lead to higher rate capability. Both HC-HT190 and HC-HT220 demonstrate a high capacity at low current densities and a high recovery capacity when the current density reverts to 0.1 A g^–1^ (272.2 and 252.9 mAh g^–1^, respectively). Although HC-HT190 demonstrated a high capacity at current densities of 0.05, 0.1, and 0.2 A g^–1^, its capacity decayed rapidly when the current density surpasses 0.4 A g^–1^. This decline becomes more pronounced when the current density reaches 1.0 A g^–1^. On the other hand, HC-HT220 demonstrated slightly lower capacity in this particular case at the low current densities (<0.2 A g^–1^) compared with HC-HT190, it showed superior capacity retention at current densities of higher than 0.2 A g^–1^. The superior performance of HC-HT220 at high current densities may be attributed to its dominant micropores with a pore size of 1–2 nm (Figure 3f) that allows fast ions transport. Considering that HC-HT220 also has a higher ICE (84.8%) than HC-HT190, which has an ICE of 79.6%, HC-HT220 is the better choice of the two (HC-HT220 and HC-HT190).

Building on the foundation of previous studies, our work takes a notable step forward by enhancing the performance of SIBs using hard carbon derived from raw biomass. As illustrated in Figure 4d and Table S7, our methodology exhibits a significant improvement in both storage capacity and ICE, validating the efficiency and potential of our approach in utilizing sustainable and renewable biomass materials for next-generation energy storage systems. The primary disadvantage of biomass-derived hard carbon, insufficient ICE, can be further addressed by chemical presodiation. Figure 4e shows further ICE improvements with different presodiation time. As demonstrated, presodiation of hard carbon for 2 min can overcome the Na loss at anode interphase during the first formation cycle. Therefore, presodiated electrodes are also used in full cell configurations.

To assess the electrochemical performance of hydrothermal hard carbon anodes in practical applications, long cycling full-cell SIBs were tested, with a Na-rich NASICON (sodium superionic conductor)-type sodium vanadium phosphate (Na_3_V_2_(PO_4_)_3_, or NVP) serving as the cathode placed against hard carbon anodes. Among various potential cathodes, NVP is distinguished as a promising material for its stable and rhombohedral structure, high voltage platform, and excellent thermal stability.^61,62^ In our well-balanced full-cell configuration, an exceptional specific capacity of 112.6 mAh g^–1^ can be achieved at the current density of 100 mA g^–1^, normalized by the active mass of the carbon anode (Figure 4f). Moreover, an advanced ICE of 94% can be achieved, suggesting the presodiation compensated the Na consumption. This performance is far superior to the full-cell battery without presodiation. As shown in Figure S7, the full cells without presodiation suffer from unstable performance and low capacity at the first few cycles with a low ICE of 48.4%. Furthermore, the presodiated HC-HT220//NVP full-cell battery demonstrates a reliable specific capacity of 99 mAh g^–1^ with a capacity retention of 88% after 10 cycles, but continuously decays to 78 mAh g^–1^ after 100 cycles (Figure S8). This capacity fading reflects underlying practical issues of storing sodium ions in hard carbon, such as electrolyte decomposition or changes in the SEI, which we aim to address in future research to improve the battery’s stability. Furthermore, coupling the NVP cathode with hard carbon anodes leads to a high energy density and stable voltage output, which are critical for practical applications that extend from portable electronics to large-scale energy storage systems.

### Chemical Characterization of the SEI and Electrochemical Dynamics

**Figure 5:**
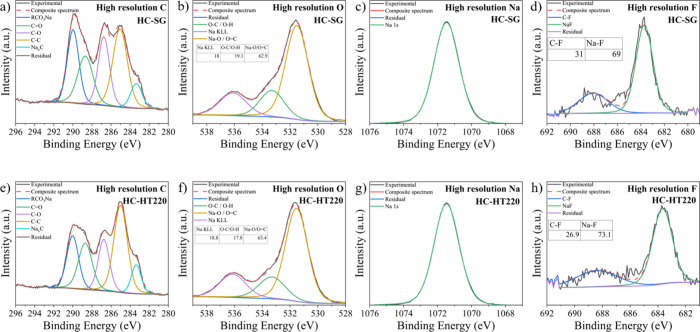
Fitted (a, e) C 1s, (b, f) O 1s, (c, g) Na 1s, and (d, h) F 1s core-level XPS spectra of HC-SG and HC-HT220 after 15 cycles at a discharge–charge rate of 0.1 A g^–1^. Each panel highlights the possible functional groups, indicating the electrolyte interaction and SEI formation during cycling.

The chemical characterization of the SEI was carried out by XPS on HC-SG and HC-HT220 after 15 discharge–charge cycles to unveil the atomic composition of the SEI layer (Figure S9). To compare the overall atomic concentrations, the cycled electrode consisting of HC-HT220 exhibited a lower oxygen content (35.8%) compared to untreated HC-SG (40.1%), which can potentially mitigate side reactions and decelerate electrolyte decomposition. In addition, we further fitted C 1s, O 1s, Na 1s, and F 1s core-level XPS spectra of the cycled samples for providing crucial information about the interface conditions. Figure 5a,e displays five distinct C 1s peaks, each representing specific carbon environments intrinsic to the diverse species observed within the SEI: (i) sodium ethylene decarbonate (Na_2_CO_3_/ROCO_2_Na) environment (∼290.0 eV), (ii) C=O (∼288.6 eV), (iii) 286.7 eV for C–O environment, (iv) C–C/C=C (∼284.8 eV), and last, (v) C atoms bound to Na atoms (∼283.1 eV). Of them, Na_2_CO_3_/ROCO_2_Na, C–O, C=O, and C–C/C=C represent organic species that originate from electrolyte decomposition, while carbon atoms bounded to sodium (Na_*x*_C) indicate the sodiated carbon underneath the SEI.^63^ By comparing the fitted C 1s high resolution spectra, HC-HT220 exhibits an increased content of Na_*x*_C and C–C in the SEI, but a lower content of RCO_3_Na and C–O compared to that of HC-SG (Table S6). Previous research has established a correlation between the chemical compositions of the cycled electrodes and their electrochemical performance, revealing that battery performance is associated with the SEI composition, particularly the presence of sodium ethylene dicarbonate and NaF.^64^ NaF is commonly formed by the reduction of solvated CF_3_SO_3_^–^.^63^ Notably, the unsatisfactory electrochemical performance of HC-SG may be attributed to its low NaF content (Figure 5d,h) coupled with a large surface area and impurity. The NaF-less SEI not only fails to protect the anode and prevent side reactions but also exacerbates the continuous electrolyte decomposition, thus compromising the battery’s stability. As discussed in the previous section, HC-SG exhibits a higher amount of heteroatoms, such as nitrogen and oxygen (Figure 3f). The heteroatoms may have participated in competing side reactions that can consume the electrolyte and active sodium ions. Previous studies stated that oxygen-containing functional groups on the anode surface can react with sodium ions to form sodium alkoxides or other organic SEI species, which may compete with the formation of NaF in the SEI.^33^ In addition, the presence of these heteroatoms can promote the adsorption of oxygen, subsequently leading to an increased concentration of sodium organic salt (Table S6) and a lower amount of NaF. In contrast, hydrothermal pretreatment effectively removed the organic impurities from switchgrass and thereafter resulted in a lower amount of heteroatoms in the pretreated hard carbon (HC-HT220). This may explain the observation of less sodium organic salt and more NaF presented on the electrodes upon cycling; however, it needs to be verified in the future. This finding underscores the role of hydrothermal pretreatment in modulating SEI by carbon surface properties. Overall, the XPS results highlight the importance of a well-formed and stable SEI in ensuring optimal cycling stability and extending battery life.

To further understand the effect of capacitive distribution on the electrochemical performance of the optimal carbon anode, i.e., HC-HT220, the CV curves were measured at sweep rates from 0.1 to 1.5 mV s^–1^ (Figure S10). The SEI formation induces a pronounced irreversibility in the CV profiles, which remains evident throughout the first three cycles (Figure S10b,c). The CV curves of HC-HT220 retain the same shape and exhibit an insignificant potential shift of redox peaks when the scan rates increase. At a scan rate of 1.5 mV s^–1^, the CV curve displays well-defined redox peaks, which corresponds to the plateau capacity observed within the lower potential range, implying the pretreatment facilitates the migration of sodium ions, leading to superior rate capability.

Furthermore, the galvanostatic intermittent titration technique (GITT) was utilized to evaluate the diffusion coefficient of sodium ions during the charge–discharge process using Fick’s second law (eq 1).
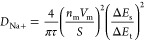
1where *D*_Na+_ is the diffusion coefficient of sodium ions, τ (s) is the pulse duration, *n*_m_ (mol) and *V*_m_ (mL mol^–1^) are the number of moles and the molar volume of the active material, *S* (cm^2^) is the contacting area between the electrode and electrolyte, Δ*E*_s_ (V) is the potential difference caused by the pulse, and Δ*E*_t_ (V) is the potential difference resulting from the charge–discharge process. Figure S11 shows the GITT profiles and the calculated results of the diffusion behavior during the second discharge and charge process. During the sodiation (Figure S11b), the calculated diffusion coefficient of HC-HT220 first slightly decreased in the sloping region and then rapidly dropped in the plateau region, followed by an increase before the cutoff potential. Similar pattern was observed in the calculated diffusion coefficient of HC-SG, but it was notably sharper than HC-HT220. The pattern manifests the different kinetics of electrochemical reactions and the sodium storage mechanism in sloping and plateau regions.^6^ The sodium ions are readily adsorbed on micropores and defective sites on the surface in the sloping region, while the slower diffusion in the plateau region demonstrates that the sodium ions are inserted into less accessible closed pores, elucidating the rapid drop during the transition of the two regions. The smaller decrease that was observed in HC-SG might be caused by the smaller interlayer spacing and fewer closed pores. During the desodiation, the calculated diffusion coefficient of HC-HT220 showed a symmetrical pattern, suggesting reversibility during charging and discharging. In contrast, HC-SG suffered from irreversible capacity loss due to the entrapment of sodium ions on the surface.

## Conclusions

In this study, we fabricated hard carbons from switchgrass using a hydrothermally assisted carbonization process. The hydrothermal pretreatment effectively removed undesired components including hemicellulose and natural impurities in switchgrass and prestabilized its structures and morphologies. As a result, hydrothermally treated hard carbons exhibited a more disordered structure, smaller surface area, more uniform micropore distribution, and reduced surface oxygen content. The optimized hard carbon (HC-HT220), characterized by a low surface area, abundant micropores, and reduced C–O functional groups, exhibited favorable properties for sodium ion accommodation. The HC-HT220 achieved a high reversible capacity of 313.4 mAh g^–1^ at 0.1 A g^–1^, a high initial Coulombic efficiency of 85%, and excellent cycling stability with 98.4% capacity retention after 100 cycles in half-battery tests. By pairing the produced anode with a suitable cathode, the electrochemical performance of the full-cell batteries can be enhanced, moving toward the development of stable, commercially viable SIBs. In conclusion, this research effectively converts low-cost and renewable switchgrass to a high-value SIB anode material, supporting the long-term sustainability of the agricultural system.

## Supporting Information Available

Morphologies of precursors and hard carbons (Figure S1); TEM images of hard carbons (Figure S2); chemical composition of precursors (Table S1); FTIR analysis of chemical composition of precursors (Table S2); yields of hydrochars and hard carbons (Table S3); structural information on hard carbons (Table S4); elemental composition of hard carbons (Table S5); fitted high-resolution C 1s XPS spectra of hard carbons (Figure S3); N2 adsorption and desorption isotherms of hard carbons (Figure S4); cycling performance of hard-carbon-derived electrodes (Figure S5); electrochemical performance among different electrolytes (Figure S6); galvanostatic discharge/charge profiles of full-cell battery without presodiation (Figure S7); Electrochemical performance of presodiated full-cell battery upon cycling (Figure S8); elemental composition of carbon anodes after cycling (Table S6); XPS surveys of carbon anodes after cycling (Figure S9); comparisons of electrochemical performance of different biomass-derived hard carbon anodes for SIBs (Table S7); cyclic voltammetry (CV) curves of carbon anodes (Figure S10); and galvanostatic intermittent titration technique (GITT) profiles and diffusion coefficients of carbon anodes (Figure S11) (PDF)
