# Supplementary material for: Hydrothermally Assisted Conversion of Switchgrass into Hard Carbon as Anode Materials for Sodium-Ion Batteries
Source: ACS Appl Mater Interfaces. 2024 May 23;16(22):28461–72. doi: 10.1021/acsami.4c02734 (PMC11163401; doi:10.1021/acsami.4c02734)
Supplement: Supplementary file 1 — am4c02734_si_001.pdf [file am4c02734_si_001.pdf]

Hydrothermally Assisted Conversion of Switchgrass into Hard Carbon as Anode Materials for Sodium-Ion Batteries

Yilin Li[1], Dawei Xia[2], Lei Tao[2], Zhiyuan Xu[1], Dajun Yu[1], Qing Jin[1,3], Feng Lin[2], Haibo Huang[1,*]

1 Department of Food Science and Technology, Virginia Tech, 1230 Washington St SW, Blacksburg, VA 24061, United States

2 Department of Chemistry, Virginia Tech, 1040 Drillfield Dr, Blacksburg, VA 24061, United States

3 School of Food and Agriculture, University of Maine, 5763 Rogers Hall, Orono, ME 04469, United States

*** Corresponding Author:**

Dr. Haibo Huang

Human & Agricultural Biosciences Bldg.1, Room 402J, 1230 Washington St. SW,

Blacksburg, Virginia 24061, USA

Tel: (540) 231-0729

Fax: (540) 231-9293

Email: huang151@vt.edu

Supporting Information

Total pages: 18

Total tables: 7

Total figures: 9

Physicochemical Properties of Materials

Scanning electron microscope (SEM)

The morphology of the switchgrass and hydrothermally treated hydrochar were visualized with a SEM (JEOL IT-500HR, JEOL, Tokyo, Japan). The switchgrass samples were coated with 10 nm platinum and visualized with high-resolution images using a Schottky field emission (FEG) electron source, while the hydrochars (i.e., HT160, HT190, and HT220) and hard carbon (i.e., HC-HT160, HC-HT190, and HC-HT220) were directly placed on conductive carbon adhesive tapes for visualization.

Transmission electron microscope (TEM)

The structures of the switchgrass and hydrothermally treated hydrochars derived hard carbon were determined using TEM. A small amount of the powder samples was uniformly dispersed in an IPA suspension with the aid of ultrasound (Sonic Dismembrator Model FB-505, Fisher Scientific, Hampton, NH, USA). The suspensions after ultrasound were transparent, light gray color. The prepared suspensions were then deposited on a carbon film supported copper grid (Carbon Film 300 Mesh, Copper, Ted Pella, Inc., Redding, CA, USA) dropwise, and air dried

overnight. The prepared samples were visualized using a TEM (JEOL S/TEM 2100, JEOL, Tokyo, Japan) at 200 kV and fast Fourier transformation (FFT) patterns.

Chemical composition analysis

The chemical composition including moisture, soluble sugars, cellulose, hemicellulose, and lignin of switchgrass and derived hydrochars was determined following the National Renewable Energy Laboratory (NREL) standard procedures NREL protocol (NREL, 2011). In brief, the switchgrass and hydrochars were hydrolyzed into monosaccharides following a two-step extraction process using a Dionex ASE 350 accelerated solvent extractor (Thermo Fisher Scientific, Waltham, MA, USA) with ultrapure water and ethanol as extraction solvents. A piece of extraction glass fiber was placed at the bottom of each extraction cell, and collection vials with lids and septa were used to collect the solvents. The remaining solids after extractions were placed into pressure tubes and mixed with 3 mL of 72% (w/w) sulfuric acid for 1 h. A set of sugar recovery standards (SRS) was prepared to correct for losses causing by the destruction of sugars during the acid hydrolysis. The SRS included D-(+)glucose, D-(+)xylose, D-(+)galactose, and D-(+)arabinose. The SRS was transferred into pressure tubes and mixed with 3 mL of 72% (w/w) sulfuric acid for 1 h. The solutions were then diluted to 4% (w/w) by adding deionized water. The tubes were autoclaved at 121 °C for 1 h. After the pressure tubes cooled to room temperature, 20 ml of the solution were extracted and neutralized to pH 5-6 by using barium carbonate. The neutralized liquor was filtered through a 0.2 μm syringe filter (Acrodisc, Cytiva Life Science, Marlborough, MA, USA) and subjected to analytical HPLC systems (1200 series, Agilent Technologies, Santa Clara, CA, USA) for sugar analysis. The ash content was determined using a muffle furnace (F62700, Barnstead|Thermolyne, UK) at 575 °C for 4 h.

Yield

The yield of produced hydrochar was calculated by the following equation:

$$Yield\ of\ hydrochar\ (\%) = \frac{mass\ of\ hydrochar\ obtained}{mass\ of\ switchgrass\ used} \times 100\ Yield\ (\%) \qquad \text{Eq. 1}$$

Fourier transform infrared spectroscopy (FTIR)

Functional groups of raw switchgrass and produced hydrochar were identified using a Fourier transform infrared spectrometer (Nicolet 8700, Thermo Fisher Scientific, Waltham, MA, USA). One to two mg of samples were mixed with 100 mg of KBr (Thermo Fisher Scientific, Waltham, MA, USA) to form a transparent thin film for light transmission.

Thermogravimetric analysis (TGA)

Thermogravimetric analysis (TGA) was used to determine the thermal stability of the switchgrass and hydrochar. The thermogravimetric analyzer (TGA Q500 V6.7, TA Instrument, New Castle, DE, USA) will continuously measure the mass change while the temperature increases from 30 °C to 900 °C with a heating rate of 5 °C min^{-1} under nitrogen atmosphere.

Carbon characterizations

Yield

The yield of produced hard carbon was calculated by the following equation:

$$Yield\ of\ hard\ carbon\ (\%) = \frac{mass\ of\ hard\ carbon\ obtained}{mass\ of\ switchgrass\ used} \qquad \text{Eq. 3}$$

The calculated yields were used to compare the conversion efficiency of switchgrass and hydrochars.

X-ray diffraction (XRD)

The interlayer spacing (d002) was calculated based on Bragg's Law (Eq. 2), and the average lateral size (L_a) and the stacking height (L_c) of the crystallite was determined using the conventional Scherrer equations (Eq. 3 and 4).

$$2d\sin(\theta) = n\lambda \qquad \text{Eq. 4}$$

where d is the lattice interplanar spacing of the crystal, θ is the x-ray incidence angle (Bragg angle), and λ is the wavelength of the characteristic x-rays.

$$L_a = 0.94\lambda/B_a\cos(\theta_a) \quad \text{Eq. 5}$$

$$L_c = 0.94\lambda/B_c\cos(\theta_c) \quad \text{Eq. 6}$$

where B_a and B_c are the width of the (100) and (002) peaks, respectively, and θ_a and θ_c are the corresponding scattering angles or peak positions.

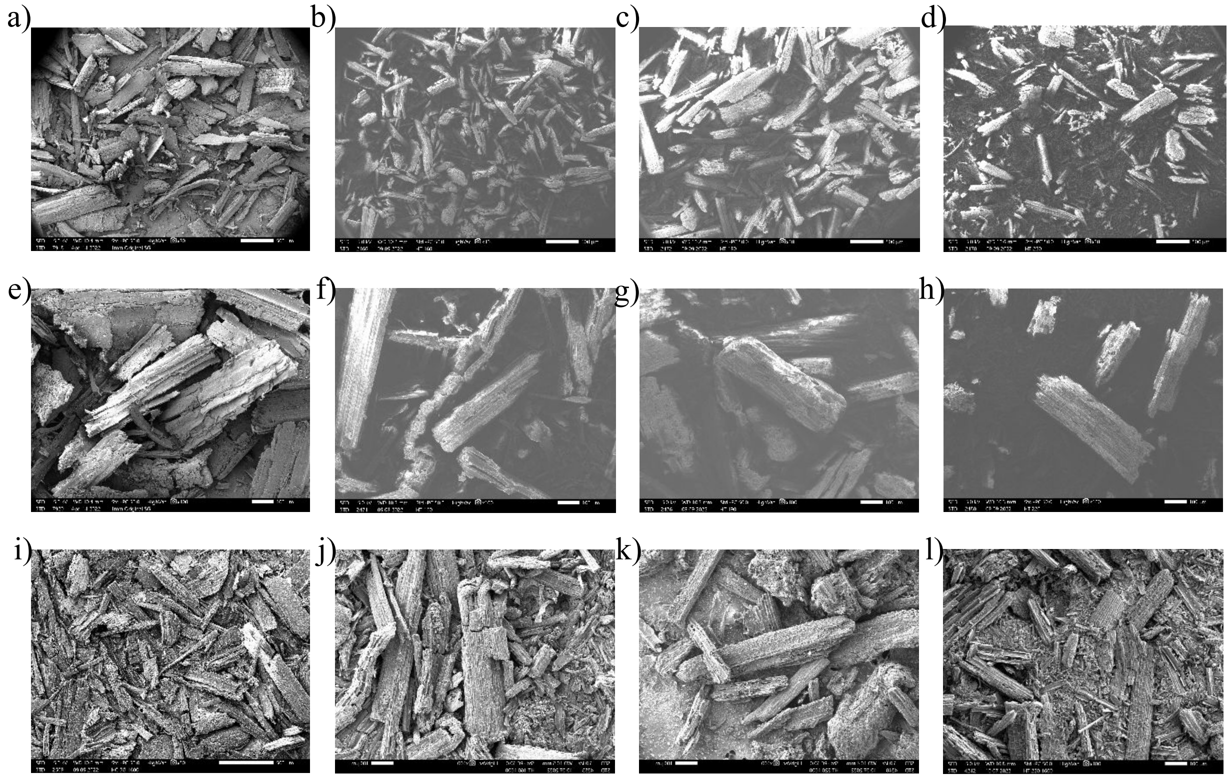

Figure S1 Morphologies of precursors and hard carbons. SEM images of a, e) raw switchgrass, b, f) HT160, c, g) HT190, d, h) HT220, (i) HC-SG, j) HC-HT160, k) HC-HT190, and l) HC-HT220.

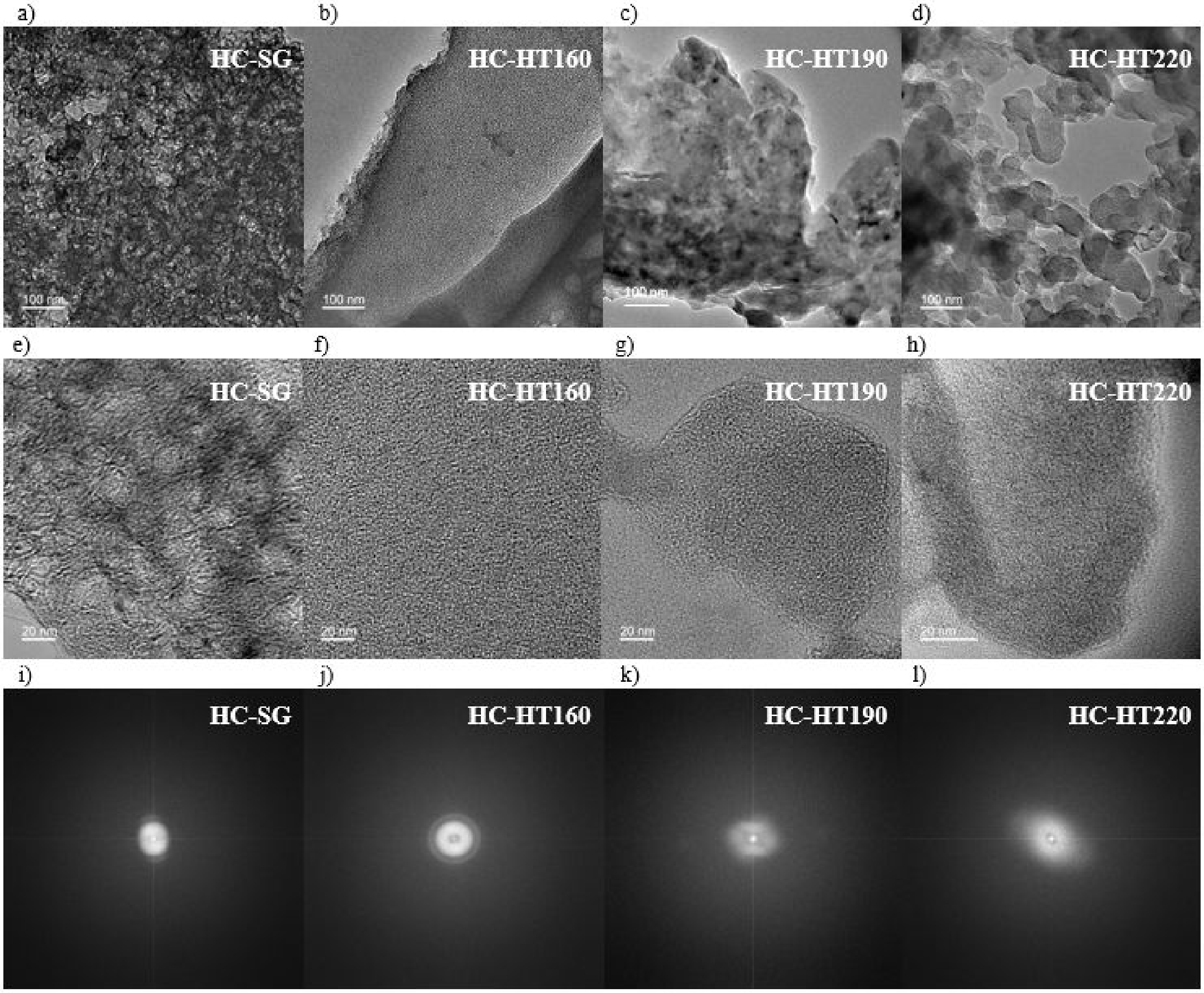

Figure S2 TEM images of a,e) HC-SG, b,f) HC-HT160, c,g) HC-HT190, and d,h) HC-HT220; and corresponding FFT images of i) HC-SG, j) HC-HT160, k) HC-HT190, and l) HC-HT220.

Table S1 Chemical composition of switchgrass and hydrochars (%, dry mass basis, WW)

Parameters	Switchgrass	HT160	HT190	HT220
Cellulose	38.51 ± 0.51	40.66 ± 3.58	43.66 ± 4.34	2.86 ± 0.73
Lignin	16.09 ± 2.78	20.58 ± 1.43	36.83 ± 1.22	77.38 ± 0.11
Xylan	26.12 ± 0.13	18.29 ± 4.16	0.48 ± 0.62	0.00 ± 0.00
Galactan	1.33 ± 0.50	0.30 ± 0.26	0.25 ± 0.42	0.17 ± 0.27

Arabinan	3.63 ± 0.36	0.78 ± 0.90	0.92 ± 0.50	0.92 ± 0.29
Mannan	0.66 ± 0.50	0.49 ± 0.81	0.65 ± 0.56	0.39 ± 0.60
Ash	2.00 ± 0.14	1.33 ± 0.14	1.14 ± 0.30	0.91 ± 0.31

Table S2 Summary of the peak location and description of the major chemical functional groups of switchgrass and hydrochars obtained from FTIR analysis.

Wavenumber (cm^{-1})	Functional group	Description	Reference
~3400	O-H stretching	Cellulose: hydroxyl group (alcohol), hemicellulose: hydroxyl group, and lignin: aromatic (phenolic) and aliphatic OH groups	5
~2900	C-H stretching	Cellulose, hemicellulose, and lignin: symmetric methyl and methylene stretching	6
~1700	C=O stretching	Cellulose and lignin: carbonyl, ester, or carboxyl	7
1600-1450	C=C stretching	Lignin: aromatic skeletal	7
1380-1240	C-O stretching	Lignin: syringyl ring	5
1060	C-O stretching	Cellulose and hemicellulose: carbonyl	7

Table S3 Yields of hydrochars and derived hard carbons.

Sample	Hydrochar (yield, %, w/w)	Hard carbon (yield, %, w/w)
SG	-	19.10
HT160	73.78	14.60
HT190	58.33	16.04
HT220	43.43	20.00

The yield of hard carbon from switchgrass is important as it is closely related to the economic viability of hydrothermal-assisted carbonization technique to produce hard carbon from switchgrass. Table S3 shows the yields of hydrochars and hard carbons obtained from raw switchgrass and hydrothermally treated hydrochars. The yields of hydrochars decrease as the temperature increased from 160 to 220 °C. This outcome is consistent with the results of the FTIR analysis, indicating that higher pretreatment temperatures facilitate the dissolution of hemicellulose and impurities into the liquid phase. Furthermore, the observation that the yield of hard carbon increased with higher pretreatment temperatures aligns consistently with the higher thermal stability exhibited by hydrochars, as discussed previously. The observed increase in hard carbon yield with higher hydrothermal pretreatment temperatures implies that the hydrothermal pretreatment pre-stabilizes the morphologies and structures, resulting in an overall increase in carbon yield during subsequent carbonization process.

Table S4 Comparison of carbon structures obtained from switchgrass and hydrochars.

Sample	θ_{100}(°)	θ_{002}(°)	L_a (Å)	L_c (Å)
HC-SG	43.34	24.67	0.78	0.98
HC-HT160	43.34	24.67	0.81	0.94
HC-HT190	43.40	24.46	0.74	0.90
HC-HT220	43.16	24.38	0.75	0.91

Table S5 Percentage of elements from XPS survey

Sample	C 1s	N 1s	O 1s
HC-SG	82.13	3.32	5.01
HC-HT160	98.73	0.07	0.87
HC-HT190	98.45	0.08	1.19
HC-HT220	98.13	0.13	1.60

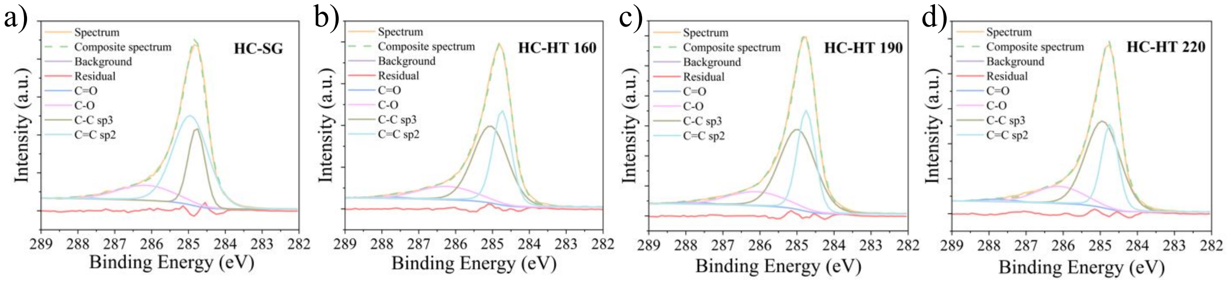

Figure S3 Fitted high-resolution C1s XPS spectra of a) HC-SG, b) HC-HT160, c) HC-HT190, and d) HC-HT220.

The XPS results revealed that the HC-SG sample contained Si while no significant amount of Si was detected in hydrothermal carbons, which aligns with the conclusion drawn

from the Raman analysis. Silicon impurities can alter the surface properties and trigger unwanted interaction with the electrolyte. The observation from XPS further reinforces the importance of removing silicon through the hydrothermal pretreatment.

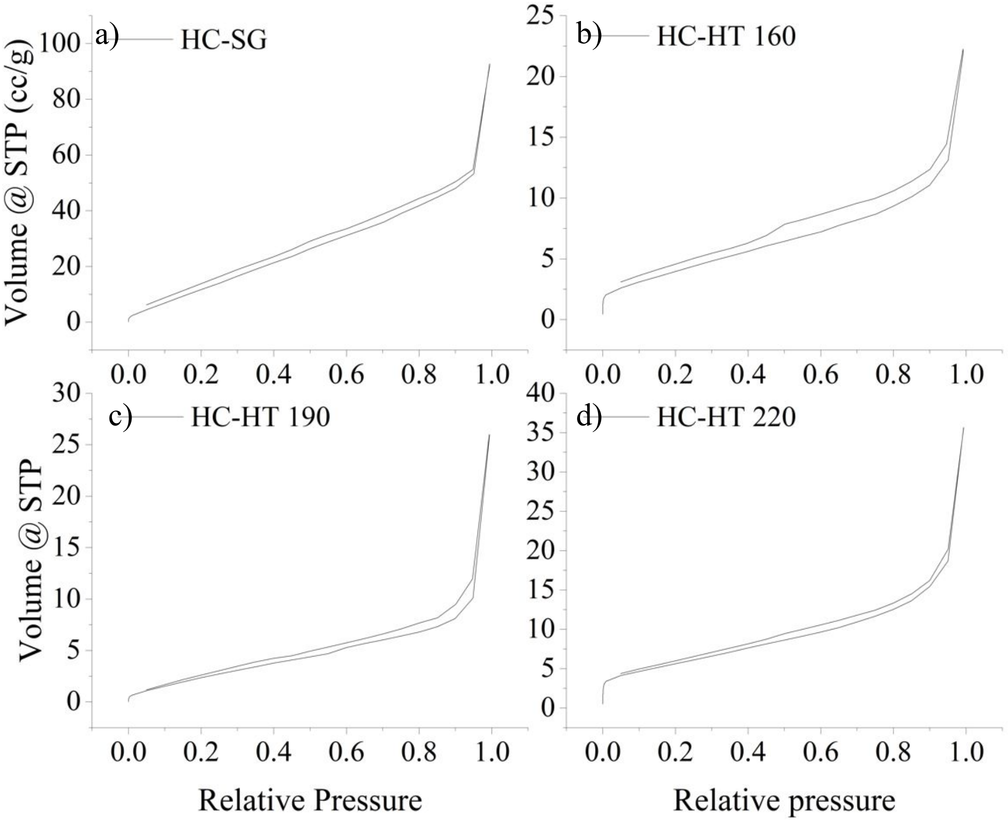

Figure S4 N_2 adsorption and desorption isotherms of a) HC-SG, b) HC-HT160, c) HC-HT190, d) and HC-HT220.

As demonstrated in Figure S4, each sample shows a different hysteresis loop, a phenomenon mainly caused by adsorption metastability or network effects.[1,2] According to the most up-to date classification from the International Union of Pure and Applied Chemistry (IUPAC), six different shapes of hysteresis loop have been recognized, and each of these six types is strongly associated with the pore structure and underlying adsorption mechanisms. The hysteresis loops observed in our carbon samples can be classified as type H3 or H4 which both

characterized by a narrow loop and no obvious adsorption saturation. This indicates that the pores of obtained hard carbons are mainly non-uniform split-like pores (micropores) formed by the accumulation of layered particles.[1] The pore-filling process is more pronounced at low p/p^0. Figure S4 shows that HC-SG has the strongest hysteresis loop effects, followed by HC-HT160. HC-HT190 and HC-HT220, conversely, exhibit weaker hysteresis loop effects, corresponding to their reduced adsorption capacity to trap sodium ions.

Electrochemical Performance

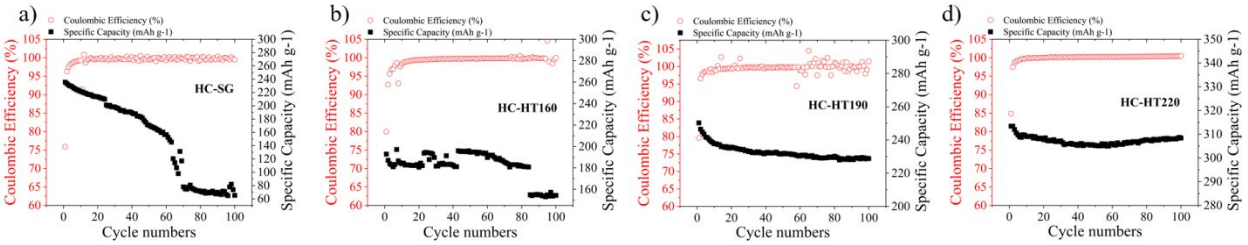

Figure S5 Comparison of the cycling performance (100 mA g^{-1}, 100 cycles) of a) HC-SG, b) HC-HT160, c) HC-HT190, d) and HC-HT220.

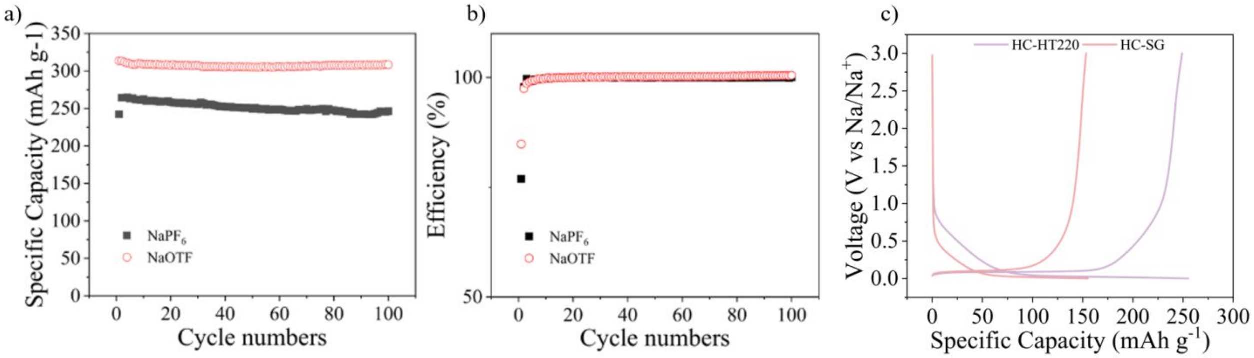

Figure S6 Comparison of electrochemical performance among different electrolytes. Comparison of a) Specific capacity and b) Coulombic efficiency of HC-HT220 using 1 M NaOTF and $NaPF_6$ in diglyme (100 mA g^{-1}, 100 cycles); c) The second cycle of galvanostatic charge/discharge (GCD) curves of HC-SG and HC-HT220 at 0.1 A g^{-1} within the potential range of 0.001-3 V versus Na/Na^+ using 1M $NaPF_6$ in EC/DMC (1:1).

While an in-depth discussion on the influence of electrolyte on SIBs is beyond the scope of this work, we noted that the electrochemical performance of carbon anodes can indeed be impacted by the composition of electrolyte, such as salt anions. Based on previous studies,[3,4] sodium trifluoromethanesulfonate (NaOTF) and sodium hexafluorophosphate ($NaPF_6$) in diglyme (G2) have been identified as the most suitable electrolytes for hard carbon anodes in SIBs. Therefore, these two ether electrolytes were selected in this work to evaluate the performance of hydrothermally treated hard carbons. The concentration of the electrolyte solution is 1 M in both cases. Upon comparison of all samples, it was observed that HC-HT series consistently reveal superior performance than HC-SG, with HC-HT220 performs the most stable performance and the highest reversibility. For $NaPF_6$ in G2 as the electrolyte, the specific charging capacity of HC-HT220 at the second cycle is 264.5 mAh g^{-1} with an ICE of 76.9% in the half-cell configuration, which shows lower electrochemical performance than that using NaOTF (Figure S6 a, b). A more significant difference is discovered during the rate test: NaOTF-based electrolytes systematically lead to low capacity at high current density compared to $NaPF_6$ counterpart.

Furthermore, we tested the electrochemical performance in ester electrolytes to align with industrial application insights. The ester electrolyte selected for the comparison was 1M $NaPF_6$ dissolved in a mixture of ethylene carbonate (EC) and DMC (dimethyl carbonate) with a volume ratio of 1:1. When diglyme is replaced by EC/DMC, HC-HT220 still exhibit a competitive performance (Figure S6c) with a specific capacity of ~255 mAh g^{-1} at the current density of 100 mA g^{-1} during the second cycle of GCD. Although the ICE decreased to 51.5%, the Columbic efficiency readily reached 98.2% in the 4th cycle. Previous studies have reported similar improvements in sodium storage performance by the use of ether-based electrolytes.[14-16]

Regardless of electrolytes, the HC-HT series consistently reveals superior performance than HC-SG, with HC-HT220 performing the most stable performance and showing the highest reversibility. To summarize, both ester-based and ether-based electrolytes are compatible with the obtained hard carbons, and the electrochemical performance using $NaPF_6$ aligns with that using NaOTF. It is thus concluded that the strategic selection of pretreatment methods and electrolyte solutions is integral for achieving stable sodium ion storage and optimizing the ICE for SIBs.

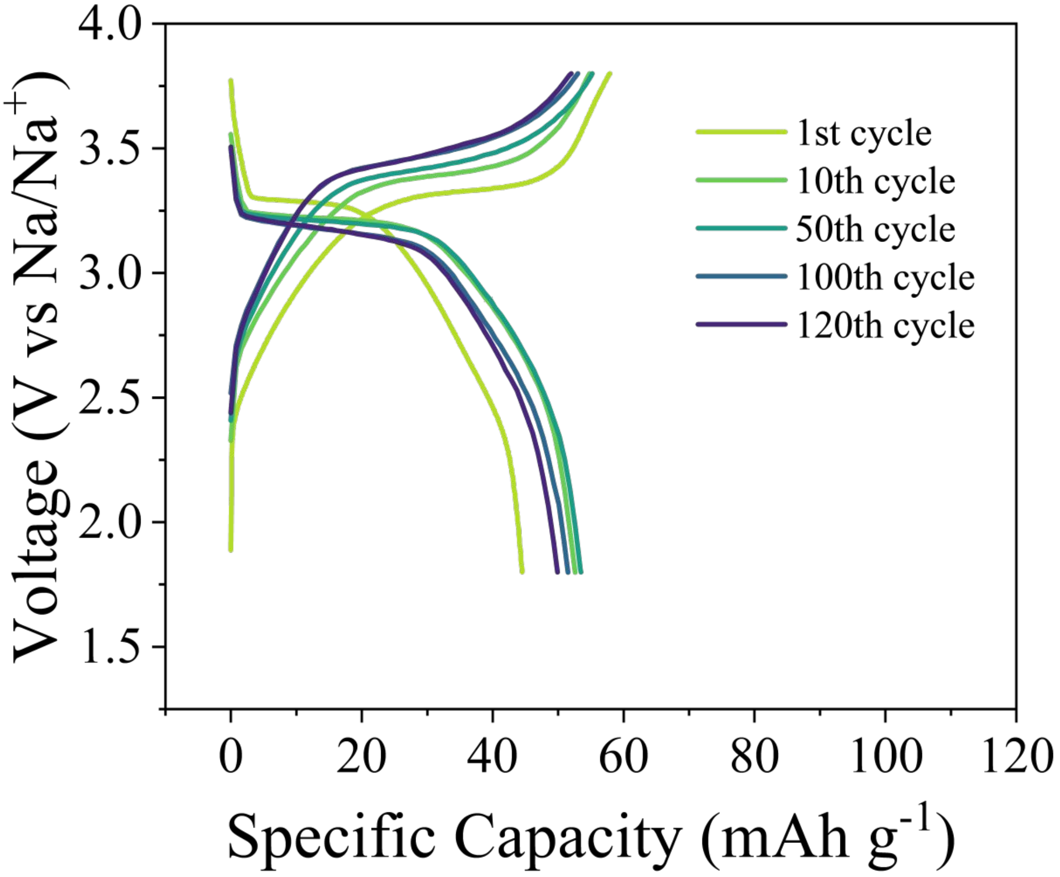

Figure S7 GCD curves of HC-HT220//NVP full-cell battery without pre-sodiation at 0.1 A g^{-1} after 1, 10, 50, 100, and 120 cycles.

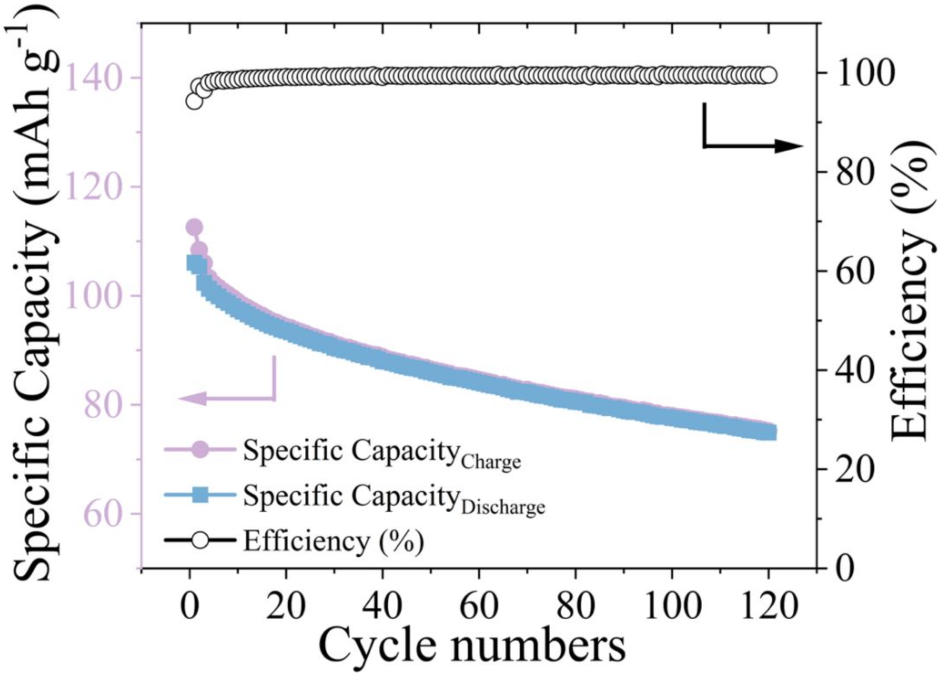

Figure S8 Specific capacity and efficiency of pre-sodiated full-cell SIBs upon cycling.

Ex-Situ X-Ray Photoelectron Spectroscopy (XPS)

The ex-situ XPS was conducted to evaluate the ionic conductivity of surface electrolyte interface (SEI) and its composition. PHI VersaProbe III photoelectron spectrometer (ULVAC-PHI, Japan) with a monochromated Al-Kα X-ray source. was used to investigate the interface conditions. Before the XPS analysis, the samples were delivered in inert sample holder affixed with double-sided tape from the argon-filled glovebox to the XPS equipment for eliminating contacting with environment.

Table S6 Percentage of functional groups from fitted high-resolution carbon.

Sample	RCO_3Na	C=O	C-O	C-C	Na_xC
HC-SG	22.3	19.5	20.6	30.1	7.5
HC-HT220	18.9	19.4	17.5	35.6	8.6

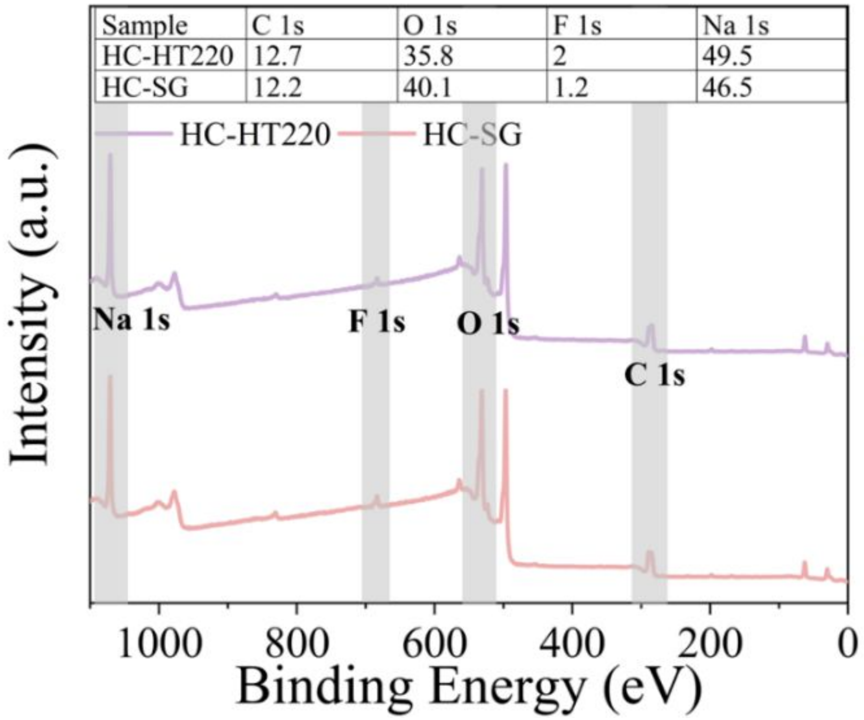

Figure S9 XPS surveys of cycled HC-HT220 and HC-SG.

Table S7 Comparisons of electrochemical performance of different biomass-derived hard carbon anodes for SIBs.

Raw material	Synthesis method	ICE (%)	$SpeCap_{charge}$ ($mAh\ g^{-1}$)	Current density ($mA\ g^{-1}$)	Reference
Corn cob	Direct pyrolysis	86	298	30	(8)
Crushed wood	Chemical pretreatment	72	260	20	(9)
Lotus seedpod	Acid washing	50	329	50	(10)5/10/2024 5:19:06 PM
Pomelo peel	Chemical activation	27	181	200	(11)

Shaddock peel	Acid washing	69	430	30	(12)
Sucrose	Hydrothermal pretreatment	83	279	30	(13)
This work	Hydrothermal pretreatment	85	313	100	-

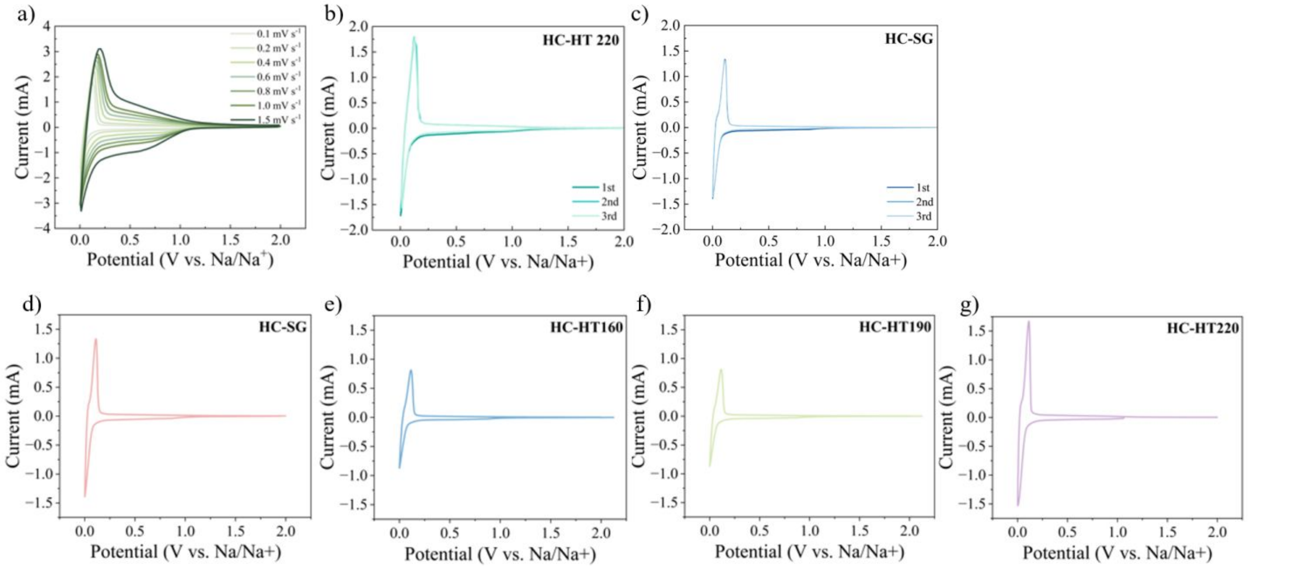

Figure S10 Kinetics analysis. CV curves of a) HC-HT220 at different scan rates of 0.1, 0.2, 0.4, 0.8, 1.0, and 1.5 mV s^{-1} (the second cycle at each scan rate), with b) the first three cycles of CV curves at 0.2 mV s^{-1}; c) the first three cycles of CV curves at 0.2 mV s^{-1} of HC-SG; the second cycle of CV curves at 0.2 mV s^{-1} of d) HC-SG, e) HC-HT160, f) HC-HT190, and g) HC-HT220.

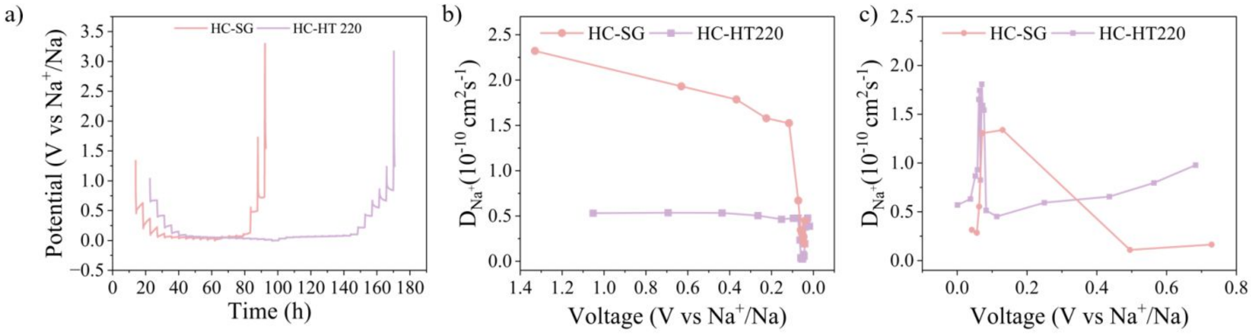

Figure S11 (a) GITT profiles of HC-SG and HC-HT220 by charging and discharging the half cells with a pulse current at 0.1 A g^{-1} for 30 min following by an interval for 1 h; Na^+ diffusion

coefficients of calculated from GITT profiles of HC-SG and HC-HT220 by (b) discharging; and (c) charging the half cells.

Reference

(1) Thommes, M.; Cychosz, K. A. Physical Adsorption Characterization of Nanoporous Materials: Progress and Challenges. *Adsorption* **2014**, *20* (2), 233–250. https://doi.org/10.1007/s10450-014-9606-z.

(2) Qi, L.; Tang, X.; Wang, Z.; Peng, X. Pore Characterization of Different Types of Coal from Coal and Gas Outburst Disaster Sites Using Low Temperature Nitrogen Adsorption Approach. *Int. J. Min. Sci. Technol.* **2017**, *27* (2), 371–377. https://doi.org/10.1016/j.ijmst.2017.01.005.

(3) Jache, B.; Adelhelm, P. Use of Graphite as a Highly Reversible Electrode with Superior Cycle Life for Sodium-Ion Batteries by Making Use of Co-Intercalation Phenomena. *Angew. Chem. Int. Ed Engl.* **2014**, *53* (38), 10169–10173. https://doi.org/10.1002/anie.201403734.

(4) Goktas, M.; Bolli, C.; Buchheim, J.; Berg, E. J.; Novák, P.; Bonilla, F.; Rojo, T.; Komaba, S.; Kubota, K.; Adelhelm, P. Stable and Unstable Diglyme-Based Electrolytes for Batteries with Sodium or Graphite as Electrode. *ACS Appl. Mater. Interfaces* **2019**, *11* (36), 32844–32855. https://doi.org/10.1021/acsami.9b06760.

(5) Md Salim, R.; Asik, J.; Sarjadi, M. S. Chemical Functional Groups of Extractives, Cellulose and Lignin Extracted from Native Leucaena Leucocephala Bark. *Wood Sci. Technol.* **2021**, *55* (2), 295–313. https://doi.org/10.1007/s00226-020-01258-2.

(6) Javier-Astete, R.; Jimenez-Davalos, J.; Zolla, G. Determination of Hemicellulose, Cellulose, Holocellulose and Lignin Content Using FTIR in Calycophyllum Spruceanum (Benth.) K. Schum. and Guazuma Crinita Lam. *PLOS ONE* **2021**, *16* (10), e0256559. https://doi.org/10.1371/journal.pone.0256559.

(7) Jaruwat, P.; Kongjao, S.; Hunsom, M. Management of Biodiesel Wastewater by the Combined Processes of Chemical Recovery and Electrochemical Treatment. *Energy Convers. Manag.* **2010**, *51* (3), 531–537. https://doi.org/10.1016/j.enconman.2009.10.018.

(8) Liu, P.; Li, Y.; Hu, Y.-S.; Li, H.; Chen, L.; Huang, X. A Waste Biomass Derived Hard Carbon as a High-Performance Anode Material for Sodium-Ion Batteries. *J. Mater. Chem. A* **2016**, *4* (34), 13046–13052. https://doi.org/10.1039/C6TA04877C.

(9) Shen, F.; Zhu, H.; Luo, W.; Wan, J.; Zhou, L.; Dai, J.; Zhao, B.; Han, X.; Fu, K.; Hu, L. Chemically Crushed Wood Cellulose Fiber towards High-Performance Sodium-Ion Batteries. *ACS Appl. Mater. Interfaces* **2015**, *7* (41), 23291–23296. https://doi.org/10.1021/acsami.5b07583.

(10) Wu, F.; Zhang, M.; Bai, Y.; Wang, X.; Dong, R.; Wu, C. Lotus Seedpod-Derived Hard Carbon with Hierarchical Porous Structure as Stable Anode for Sodium-Ion Batteries. *ACS Appl. Mater. Interfaces* **2019**, *11* (13), 12554–12561. https://doi.org/10.1021/acsami.9b01419.

(11) Hong, K.; Qie, L.; Zeng, R.; Yi, Z.; Zhang, W.; Wang, D.; Yin, W.; Wu, C.; Fan, Q.; Zhang, W.; Huang, Y. Biomass Derived Hard Carbon Used as a High Performance Anode Material for Sodium Ion Batteries. *J. Mater. Chem. A* **2014**, *2* (32), 12733–12738. https://doi.org/10.1039/C4TA02068E.

(12) Sun, N.; Liu, H.; Xu, B. Facile Synthesis of High Performance Hard Carbon Anode Materials for Sodium Ion Batteries. *J. Mater. Chem. A* **2015**, *3* (41), 20560–20566. https://doi.org/10.1039/C5TA05118E.

(13) Li, Y.; Xu, S.; Wu, X.; Yu, J.; Wang, Y.; Hu, Y.-S.; Li, H.; Chen, L.; Huang, X. Amorphous Monodispersed Hard Carbon Micro-Spherules Derived from Biomass as a

High Performance Negative Electrode Material for Sodium-Ion Batteries. *J. Mater. Chem. A* **2014**, *3* (1), 71–77. https://doi.org/10.1039/C4TA05451B.

(14) Su, D.; Kretschmer, K.; Wang, G. Improved Electrochemical Performance of Na-Ion Batteries in Ether-Based Electrolytes: A Case Study of ZnS Nanospheres. *Advanced Energy Materials* **2016**, *6* (2), 1501785. https://doi.org/10.1002/aenm.201501785.

(15) Hu, M.; Zhou, H.; Gan, X.; Yang, L.; Huang, Z.-H.; Wang, D.-W.; Kang, F.; Lv, R. Ultrahigh Rate Sodium Ion Storage with Nitrogen-Doped Expanded Graphite Oxide in Ether-Based Electrolyte. *J. Mater. Chem. A* **2018**, *6* (4), 1582–1589. https://doi.org/10.1039/C7TA09631C.

(16) Li, K.; Zhang, J.; Lin, D.; Wang, D.-W.; Li, B.; Lv, W.; Sun, S.; He, Y.-B.; Kang, F.; Yang, Q.-H.; Zhou, L.; Zhang, T.-Y. Evolution of the Electrochemical Interface in Sodium Ion Batteries with Ether Electrolytes. *Nat Commun* **2019**, *10* (1), 725. https://doi.org/10.1038/s41467-019-08506-5.
